# Anti-Photoaging Effects of Antioxidant Peptide from Seahorse (*Hippocampus abdominalis*) in In Vivo and In Vitro Models

**DOI:** 10.3390/md22100471

**Published:** 2024-10-14

**Authors:** Fengqi Yang, Yang Yang, Dandan Xiao, Poongho Kim, Jihee Lee, You-Jin Jeon, Lei Wang

**Affiliations:** 1 Department of Marine Life Sciences, Jeju National University, Jeju 63243, Republic of Korea; yfq426@naver.com (F.Y.); dandanxiao98@gmail.com (D.X.); 2 State Key Laboratory of Marine Food Processing & Safety Control, College of Food Science and Engineering, Ocean University of China, Qingdao 266404, China; yangy72000@163.com; 3 South Sea Fisheries Research Institute, National Institute of Fisheries Science, Yeosu 59780, Republic of Korea; phkim1@korea.kr (P.K.); easyhee2@korea.kr (J.L.); 4 Sanya Oceanographic Institution, Ocean University of China, Sanya 572024, China

**Keywords:** *Hippocampus abdominalis*, UVB irradiation, antioxidant peptide, photoprotective

## Abstract

Overexposure to ultraviolet (UV) radiation can lead to photoaging, which contributes to skin damage. The objective of this study was to evaluate the effects of an antioxidant peptide (SHP2) purified from seahorse (*Hippocampus abdominalis*) alcalase hydrolysate on UVB-irradiated skin damage in human keratinocyte (HaCaT) and human dermal fibroblast (HDF) cells and a zebrafish model. The data revealed that SHP2 significantly enhanced cell viability by attenuating apoptosis through the reduction of intracellular reactive oxygen species (ROS) levels in UVB-stimulated HaCaT cells. Moreover, SHP2 effectively inhibited ROS, improved collagen synthesis, and suppressed the secretion of matrix metalloproteinases (MMPs) in UVB-irradiated HDF cells. SHP2 restored the protein levels of HO-1, Nrf2, and SOD, while decreasing Keap1 expression in UVB-treated HDF, indicating stimulation of the Keap1/Nrf2/HO-1 signaling pathway. Furthermore, an in vivo study conducted in zebrafish confirmed that SHP2 inhibited photoaging by reducing cell death through the suppression of ROS generation and lipid peroxidation. Particularly, 200 µg/mL of SHP2 exerted a remarkable anti-photoaging effect on both in vitro and in vivo models. These results demonstrate that SHP2 possesses antioxidant properties and regulates skin photoaging activities, suggesting that SHP2 may have the potential for use in the development of cosmetic products.

## 1. Introduction

UVB radiation is a well-known environmental factor that causes skin aging. Exposure to UVB radiation induces DNA damage, cell apoptosis, and oxidative stress, resulting in the upregulation of metalloproteinases (MMPs) and degradation of the extracellular matrix (ECM) [1,2]. UVB radiation can cause skin photoaging by inducing the generation of ROS and disrupting the balance of ECM formation and degradation, leading to collagen degradation, which is characterized by wrinkle formation, sagging, and pigmentation [3,4]. Therefore, studying the interactions between UVB radiation, MMPs, and ECM degradation, as well as maintaining the ROS balance, is crucial for discovering protective ingredients against photoaging and maintaining skin health.

Marine bioresources encompass various organisms living in the ocean, including algae, shellfish, fish, mollusks, and marine microorganisms [5]. In recent years, marine bioresources have received significant attention owing to their rich bioactive compound content, such as bioactive peptides. These peptides have attracted considerable interest due to their diverse biological activities, including antioxidant, anti-inflammatory, anticancer, and antimicrobial properties [6]. They hold immense potential for application in cosmetics, functional foods, pharmaceuticals, and other fields. Marine-derived bioactive peptides are known for their superior safety profiles, intestinal bioavailability, and homology with human collagen [7]. Their unique structural features and bioavailability make them attractive candidates for incorporation into cosmetics and development as functional ingredients.

Seahorses have been used in traditional medicine due to their antioxidant, anti-arthritis, and anti-fatigue properties [8,9]. Among the various seahorse species, *H. abdominalis*, commonly known as the big-belly seahorse, is renowned for its large size, capable of growing up to 35 cm in length, as well as its unique characteristics, such as pale coloration and smooth skin [10,11]. Additionally, *H. abdominalis* is notable for its rich protein content, featuring a high ratio of essential amino acids, including aromatic and heterocyclic amino acids, as well as acidic amino acids [12]. Peptides extracted from seahorse tissues have attracted the interest of researchers because of their biological properties and potential therapeutic effects. These peptides have long been studied for their pharmacological effects and are valuable resources for biomedical research and drug development. A previous study identified *H. abdominalis* peptide sequences and investigated their alkyl radical scavenging activity [13]. Additionally, the *H. abdominalis* peptide demonstrated significant protective effects against AAPH-induced oxidative damage in Vero cells and zebrafish in a previous study [10]. To further explore the bioactivity of SHP2, we evaluated its potential antioxidant and anti-photoaging effects in vitro using HaCaT and HDF cells, as well as in vivo in zebrafish. This study aimed to elucidate the potential of SHP2 in mitigating UVB-induced skin damage and to investigate its possible applications in cosmetic product development.

## 2. Results

### 2.1. Protective Effect of SHP2 against UVB-Irradiated HaCaT Cell Skin Damage

**Figure 1:**
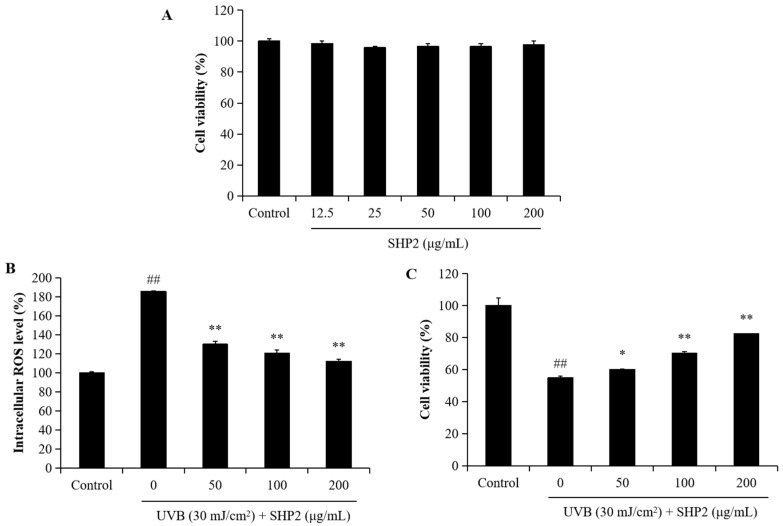
SHP2 protects HaCaT cells against UVB-induced photodamage. (**A**) Cytotoxicity of SHP2 on HaCaT cells; (**B**) intracellular ROS scavenging effect of SHP2 in UVB-irradiated HaCaT cells; (**C**) protective effect of SHP2 against UVB-induced cell death in HaCaT cells. The experiments were conducted in triplicate and the data are expressed as the mean ± SE. * *p* < 0.05, ** *p* < 0.01 as compared to the UVB-irradiated group and ## *p* < 0.01 as compared to the control group.

**Figure 2:**
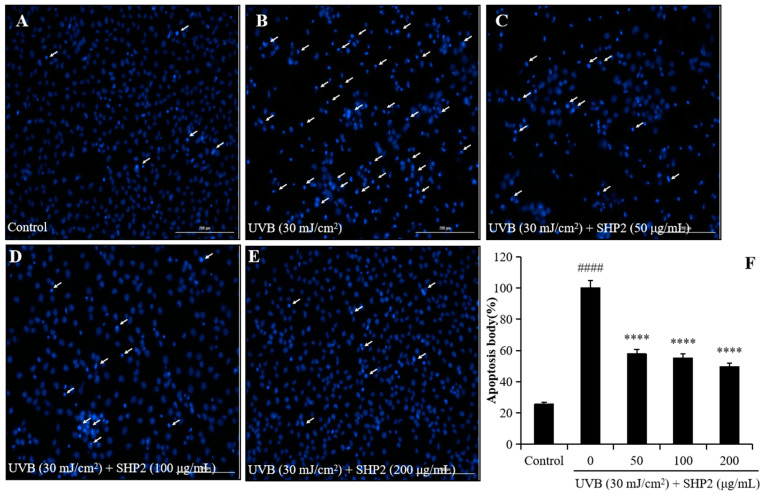
SHP2 protects HaCaT cells against UVB-induced apoptosis. (**A**) Morphology of the normal cells; (**B**) morphology of the cells irradiated by UVB; (**C**) morphology of the cells treated with 50 μg/mL SHP2 and irradiated by UVB; (**D**) morphology of the cells treated with 100 μg/mL SHP2 and irradiated by UVB; (**E**) morphology of the cells treated with 200 μg/mL SHP2 and irradiated by UVB; (**F**) quantification of apoptotic cells. The apoptotic body formation was evaluated by Hoechst 33342 staining assay. White arrows indicate apoptotic bodies. **** *p* < 0.0001 as compared to the UVB-irradiated group and #### *p* < 0.0001 as compared to the control group.

UV exposure induces oxidative damage and generates intracellular ROS, leading to cell death. Previous studies have demonstrated that *H. abdominalis* peptides effectively scavenge alkyl radicals and have an antioxidant effect [13,14]. In the present study, we investigated the effects of SHP2 on UVB-induced oxidative stress and apoptosis in HaCaT cells. We analyzed the effects of different concentrations of SHP2 on the viability of HaCaT cells. As depicted in Figure 1A, concentrations of SHP2 ranging from 12.5 to 200 µg/mL did not significantly affect HaCaT cell viability, indicating that SHP2 is not toxic to the cells. Next, we evaluated the effect of UVB radiation on intracellular ROS levels and determined whether SHP2 inhibited the increase in ROS levels induced by UVB radiation. As shown in Figure 1B, UVB radiation significantly increased intracellular ROS levels in HaCaT cells, whereas SHP2 reduced the amount of UVB-induced ROS in a concentration-dependent manner, indicating its antioxidant properties. Furthermore, as illustrated in Figure 1C, UVB radiation significantly decreased the viability of UVB-irradiated HaCaT cells, whereas SHP2 increased it in a dose-dependent manner. Additionally, as shown in Figure 2, the cells exhibited significant damage due to UVB radiation, with an increased intensity and clustering of blue spots, suggesting cell stress or death. In contrast, SHP2 significantly inhibits apoptosis, particularly at high concentrations. These results suggest that SHP2 provides a substantial protection against UVB-induced damage. Overall, SHP2 protects HaCaT cells against UVB-induced photoaging by inhibiting intracellular ROS and apoptosis.

### 2.2. Protective Effect of SHP2 against UVB-Induced HDF Cell Model

**Figure 3:**
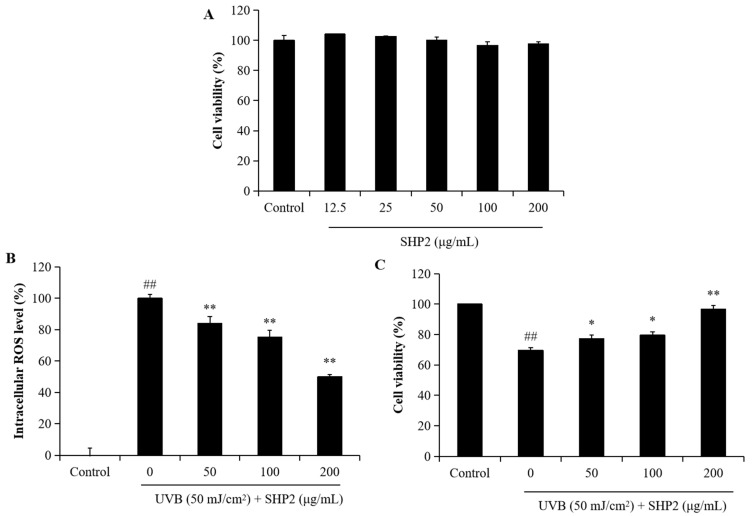
SHP2 protects HDF cells against UVB-induced damage. (**A**) Cytotoxicity of SHP2 on HDF cells; (**B**) intracellular ROS scavenging effect of SHP2 in UVB-irradiated HDF cells; (**C**) protective effect of SHP2 against UVB-induced cell death in HDF cells. The experiments were conducted in triplicate and the data are expressed as the mean ± SE. * *p* < 0.05, ** *p* < 0.01 as compared to the UVB-irradiated group and ## *p* < 0.01 as compared to the control group.

UV irradiation can stimulate the expression of MMPs, inducing oxidative damage to cells and ECM degradation. Collagen, a major component of the ECM, is highly susceptible to degradation when the level of MMP collagenases increases, which is a key cause of skin aging [15]. To further demonstrate the protective benefits of SHP2 against UVB-induced skin damage, we evaluated its photoprotective effects on HDF cells by assessing the ROS levels, collagen synthesis, and MMP expression. As shown in Figure 3A, SHP2 showed no toxicity on HDF cells at concentrations ranging from 12.5 to 200 µg/mL. In Figure 3B, intracellular ROS levels sharply increased following UVB radiation, but SHP2 treatment groups ranging from 50 to 200 µg/mL showed significantly lower values compared to that in the model group. Concurrently, cell viability in the SHP2 treatment groups steadily increased, indicating protection against UVB-triggered damage in HDF (Figure 3C).

**Figure 4:**
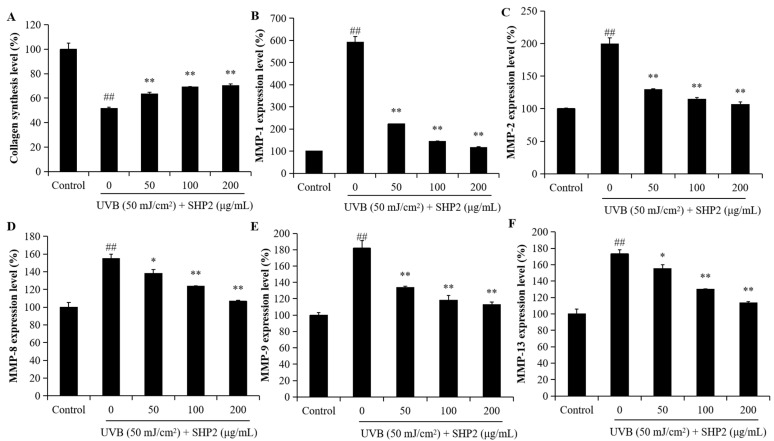
SHP2 improves collagen synthesis and inhibits MMPs’ expression in UVB-irradiated HDF cells. (**A**) Collagen contents in UVB-irradiated HDF cells; (**B**) MMP-1 expression levels in UVB-irradiated HDF cells; (**C**) MMP-2 expression levels in UVB-irradiated HDF cells; (**D**) MMP-8 expression levels in UVB-irradiated HDF cells; (**E**) MMP-9 expression levels in UVB-irradiated HDF cells; (**F**) MMP-13 expression levels in UVB-irradiated HDF cells. The experiments were conducted in triplicate and the data are expressed as the mean ± SE. * *p* < 0.05, ** *p* < 0.01 as compared to the UVB-irradiated group and ## *p* < 0.01 as compared to the control group.

As shown in Figure 4A, collagen levels decreased following UVB irradiation. However, co-treatment with SHP2 at concentrations ranging from 50 to 200 µg/mL significantly restored the collagen synthesis level. This result indicated that treatment with SHP2 enhanced collagen production, contributing to its anti-wrinkle effect. Additionally, Figure 4B–F shows the high MMP levels in UVB-irradiated HDF. However, SHP2 significantly reduced the expression levels of MMP-1,2,8,9, and 13. In particular, the reduction in MMP-8 and MMP-13 stimulated by UVB exposure was restrained in a concentration-dependent manner when HDF cells were incubated with SHP2. SHP2-treated groups exhibited a downward trend in MMPs’ expression, indicating a reduction in MMPs’ activity, which may be beneficial for maintaining ECM integrity. These results demonstrate that SHP2 can inhibit the generation of MMPs in the HDF cells induced by UVB radiation and stimulate the synthesis of collagen proteins, providing evidence for SHP2 as a potential cosmetic ingredient.

**Figure 5:**
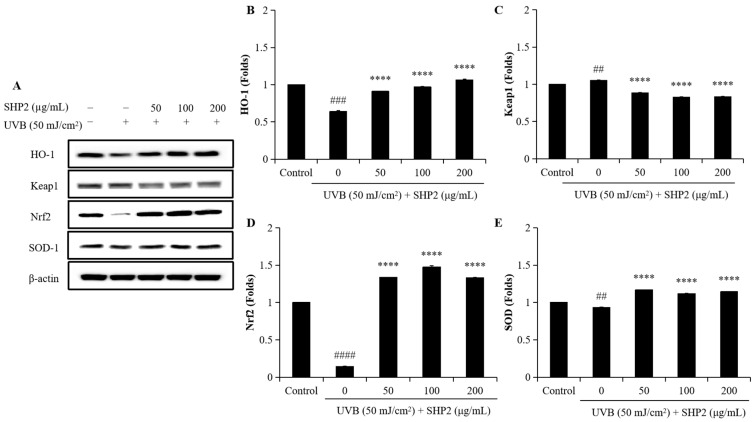
Protective effects of SHP2 on Keap1/Nrf2/HO-1 signaling pathway in UVB-irradiated HDF cells. (**A**) Western blot bands; (**B**) HO-1 protein expression; (**C**) Keap1 protein expression; (**D**) Nrf2 protein expression; (**E**) SOD protein expression. The experiments were conducted in triplicate and the data are expressed as the mean ± SE. **** *p* < 0.0001 as compared to the UVB-irradiated group and ## *p* < 0.01, ### *p* < 0.001, #### *p* < 0.0001 as compared to the control group.

To evaluate the protective effects of SHP2 against UVB-induced photoaging in HDF cells, we analyzed the expression levels of Keap1, Nrf2, HO-1, and SOD. As shown in Figure 5, UVB treatment significantly decreased the protein levels of HO-1, Nrf2, and SOD, which are the key proteins involved in cellular defense against oxidative stress, while increasing Keap1 expression compared to the control group. Supplementation with 50 µg/mL to 200 µg/mL of SHP2 significantly restored the levels of HO-1, Nrf2, and SOD. Additionally, Keap1 expression significantly decreased following SHP2 treatment. These results further support the hypothesis that SHP2 exerts protective effects against UVB-induced oxidative damage and photoaging in HDF cells by modulating the Keap1/Nrf2/HO-1 signaling pathway.

### 2.3. Protective Effect of SHP2 in UVB-Irradiated Zebrafish Model

**Figure 6:**
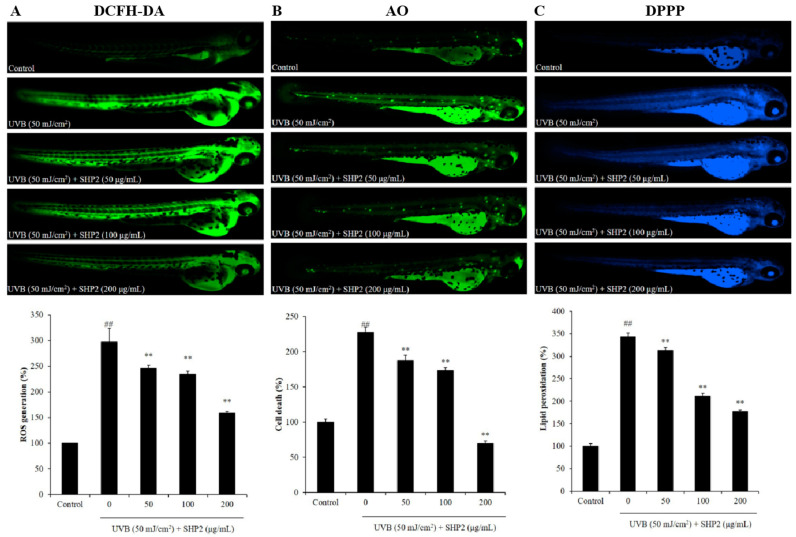
SHP2 protects zebrafish against UVB-induced oxidative stress in the zebrafish. (**A**) ROS generation in zebrafish; (**B**) cell death level in zebrafish; (**C**) lipid peroxidation level in zebrafish. ROS, cell death, and lipid peroxidation levels were measured by Image J software (v.1.8.0). The data are expressed as means ± SE. ** *p* < 0.01 as compared to the UVB-treated group and ## *p* < 0.01 as compared to the control group.

Previous studies have suggested that because of the genetic and organ system similarities of zebrafish to humans, ease of cultivation, high throughput, and low cost, zebrafish can be used as an in vivo model to measure UVB irradiation-induced cell death, intracellular ROS levels, and lipid peroxidation to evaluate the photoprotective activities of active substances [3,16,17]. In this study, we explored the in vivo photoprotective activity of SHP2 in UVB-irradiated zebrafish. As shown in Figure 6, after UVB exposure, there was a significant increase in ROS generation, cell death, and lipid peroxidation, leading to elevated oxidative stress, cytotoxicity, and oxidative damage caused to the cell membrane. However, SHP2 significantly inhibited ROS production, cell death, and lipid peroxidation in UVB-exposed zebrafish across all the SHP2 treatment groups. These results imply that SHP2 prevents photoaging in vivo in zebrafish.

## 3. Discussion

Under normal circumstances, the human body maintains a balance between the antioxidant and oxidative defenses. However, when endogenous factors, such as inflammation and metabolism, or exogenous factors, such as ultraviolet (UV) radiation, lead to the excessive production of ROS, this balance is disrupted. UV radiation can decrease cellular antioxidant levels and stimulate the production of intracellular ROS, leading to cell death, and is considered a primary environmental factor contributing to skin aging. The skin aging induced by UV radiation is commonly referred to as photoaging [18,19].

Antioxidant peptides have wide applications in various fields such as cosmetics, food, and pharmaceuticals. Because of their rich bioactivity, many researchers have focused on the extraction of new peptides from plants, terrestrial organisms, and marine animals. Marine peptides exhibit an extensive range of biological activities, including antioxidant, anti-aging, anti-hypertensive, anti-fatigue, immunomodulatory, and other functions [20,21]. The seahorse is a precious marine animal belonging to the Syngnathidae family. Reports suggest that the peptides extracted from *H. abdominalis* possess anti-fatigue, antihypertensive, and antioxidant effects [8,22,23]. However, the photoprotective role of *H. abdominalis* antioxidant peptides still needs to be evaluated. Traditional in vitro experiments such as free-radical scavenging and metal ion chelation assays cannot easily simulate the natural physiological environment of the human body. Therefore, we conducted the experiments using two types of skin cells in vitro and a zebrafish model in vivo to verify the cosmeceutical effects of SHP2 from multiple perspectives, including its antioxidant, anti-apoptotic, and anti-photoaging effects.

UVB radiation can induce the excessive production of free radicals in the human body, and an overabundance of ROS can lead to skin aging, resulting in dullness, wrinkles, and dysfunction by triggering cell apoptosis [24]. Antioxidant peptides possess the ability to scavenge free radicals, rapidly absorb them, exhibit excellent stability, and effectively delay skin aging. SHP2 significantly reduced ROS levels in a dose-dependent manner and inhibited UVB-induced apoptosis in HaCaT and HDF cells, thereby demonstrating photoprotective effects. The antioxidant activity of protein hydrolysates is highly dependent on the sequence, molecular weight, and composition of peptides. SHP2 is a low-molecular-weight peptide (913 Da) containing proline and tryptophan at the carboxyl terminus [13], which contributes to its bioactivity and anti-photoaging effects.

UVB radiation can cause wrinkles and skin damage by stimulating MMP expression and inducing the excessive production of intracellular ROS. Increased skin enzyme activity results in protein hydrolysis in the ECM [25]. Furthermore, UVB stimulation leads to the overexpression of MMP-1, resulting in the degradation of the type I and III collagen as well as the upregulation of MMP-2 and MMP-9, contributing to ECM degradation. Prolonged exposure to UV radiation accelerates the loss of collagen, reducing the synthesis of elastic fiber proteins, which are the major causes of wrinkling and skin aging [26,27]. SHP2 significantly reduced cell apoptosis, enhanced collagen synthesis, and inhibited MMP 1, 2, 8, 9, and 13 expression in a dose-dependent manner. Therefore, SHP2 demonstrated strong photoprotective activity. SHP2 also exhibited an anti-skin aging effect by downregulating ROS production and preventing the UVB-induced apoptosis of HaCaT cells. Experimental evidence suggests that some antioxidant peptides can confer anti-aging effects by enhancing the type I collagen expression and reducing that of MMP-1 [28]. SHP2 inhibited MMP expression in UVB-irradiated HDF, thereby suppressing collagen degradation, indicating its ability to promote the formation of elastic fiber proteins. These data indicated that SHP2 has photoprotective activity and the potential to delay skin aging.

The Keap1/Nrf2/HO-1 signaling pathway was analyzed by Western blotting to investigate the protective effects of SHP2 against UVB-induced photoaging in HDF cells. UVB exposure disrupted this pathway, as evidenced by the downregulation of HO-1, Nrf2, and SOD, and the upregulation of Keap1 expression. Treatment with SHP2 successfully restored the protein levels of HO-1, Nrf2, and SOD while reducing Keap1 expression, thus enhancing the antioxidant defense of cells and maintaining cellular homeostasis under UVB radiation, indicating SHP2’s potential anti-photoaging effects. Nrf2 is a key transcription factor that regulates the expression of antioxidant and cytoprotective genes. Its activation induces the expression of enzymes, such as SOD and HO-1, which neutralize ROS, a major contributor to oxidative stress and photoaging [24]. Additionally, SHP2’s ability to downregulate Keap1, which targets Nrf2 for degradation, facilitates its protective action against oxidative damage. As oxidative damage is the primary cause of photoaging, these findings suggest that SHP2’s modulation of the Keap1/Nrf2/HO-1 pathway holds therapeutic potential for preventing or mitigating UVB-induced photoaging.

Although cell and rodent models have been widely used for activity assessment, non-rodent models, such as zebrafish, offer many advantages, e.g., low maintenance costs, small size, and short life cycle, which can effectively improve experimental efficiency [29]. Moreover, owing to their transparency, zebrafish allow the use of fluorescent probes to detect cell death, ROS generation, and lipid peroxidation, making them ideal for high-throughput drug screening [30]. In vivo results indicated that SHP2 significantly inhibits ROS generation in zebrafish, effectively reducing in vivo UVB-induced photoaging by UVB stimulation through decreasing lipid peroxidation and cell death. These results suggest that SHP2 has tremendous potential for use in the cosmetics industry and merits further exploration.

To further understand the protective role of SHP2 against UVB-induced skin damage, it is essential to focus on the mechanisms of DNA damage. Recent studies highlight the critical role of DNA damage in UVB-induced skin injury, which, if unrepaired, increases skin cancer risk. For example, trigonelline prevents oxidative DNA damage by modulating the PI3K-Akt-Nrf2 signaling pathway in HDF cells and mice [31]. Similarly, asperyellone protects HDF cells from UVB-induced oxidative stress and DNA damage, reducing apoptosis and maintaining cellular integrity [32]. These findings emphasize the importance of addressing UVB-induced DNA damage to prevent the survival and proliferation of DNA-damaged cells, which may reduce the risk of developing skin cancer. Future studies will explore the potential of SHP2 in mitigating UVB-induced DNA damage to further confirm its protective effects against photoaging.

## 4. Materials and Methods

### 4.1. Materials

The dimethyl sulfoxide (DMSO), acridine orange (AO), 3-(4-5-dimethyl-2yl)-2-5-diphynyltetrasolium bromide (MTT), 2, 7-dichlorodihydroflurescin diacetate (DCFH-DA), 1,3-Bis (diphenylphosphino) propane (DPPP) phosphate-buffered saline (PBS), human matrix metalloproteinases (MMP)-1, 2, 8, 9, and 13, and Enzyme-Linked Immunosorbent Assay (ELISA) kit were purchased from Sigma Co., Ltd. (St. Louis, MO, USA). Dulbecco’s modified Eagle’s medium (DMEM), penicillium/streptomycin (P/S), fetal bovine serum (FBS), and trypsin-EDTA were purchased from Gibco BRL (Life Technologies, Burlington, ON, Canada). The primary antibodies, anti-heme oxygenase 1 (HO-1) (rabbit monoclonal antibody, Cat. #70081S), anti-Nrf2 (rabbit monoclonal antibody, Cat. #12721S), and anti-Keap1 (rabbit monoclonal antibody, Cat. #8047S), were purchased from Cell Signaling Technology (Beverly, MA, USA). Anti-Superoxide dismutase 1 (SOD1) (mouse monoclonal antibody, Cat. #SC-17767) and β-actin (mouse monoclonal antibody, Cat. #SC-47778) were obtained from Santa Cruz Biotechnology (Santa Cruz, CA, USA). The secondary antibodies, including the anti-rabbit IgG, HRP-linked antibody (Cat. #7074S) and anti-mouse IgG, HRP-linked antibody (Cat. #7076S), were obtained from Cell Signaling Technology (Beverly, MA, USA). 

### 4.2. Purification of Bioactive Peptides from H. abdominalis 

The extraction and purification of peptides from *H. abdominalis* alcalase hydrolysate (HA) were performed according to previously reported methodologies [13]. To isolate the antioxidant peptides, a process combining ultrafiltration and Sephadex purification was employed, followed by enzyme-assisted hydrolysis. This process resulted in the separation of three distinct molecular weight fractions: HA-I (>10 kDa), HA-II (5–10 kDa), and HA-III (<5 kDa). Among these fractions, HA-III, which exhibited the strongest bioactivity, was selected for further purification of the antioxidant peptides using a Sephadex G10 column. Finally, three peptides were identified: GIIGPSGSP (Glycine–Isoleucine–Isoleucine–Glycine–Proline–Serine–Glycine–Serine–Proline: SHP1), IGTGIPGIW (Isoleucine–Glycine–Threonine–Glycine–Isoleucine–Proline–Glycine–Isoleucine–Tryptophan: SHP2), and QIGFIW (Glutamine–Isoleucine–Glycine–Phenylalanine–Isoleucine–Tryptophan: SHP3). Previous studies have shown that IGTGIPGIW possesses strong free radical scavenging properties in both in vivo and in vitro models [10]. Additionally, Kim [11] showed that among the peptides extracted from seahorses, IGTGIPGIW had a relatively stronger alkyl radical scavenging capability compared to that of GIIGPSGSP and QIGFIW. Consequently, we selected SHP2 to conduct subsequent studies on UVB-caused skin damage, both in vivo and in vitro.

### 4.3. Cell Culture and Cell Viability Analysis

HaCaT cells were cultured in DMEM supplemented with 10% FBS and 1% streptomycin/penicillin. HDF cells were cultured in DMEM mixed with F-12 media supplemented with 10% FBS and 1% streptomycin/penicillin. The cultures were maintained at 37 °C in a humidified incubator with 5% CO_2_. HaCaT and HDF cells were purchased from the Korean Cell Line Bank, subcultured, and seeded at concentrations of 1 × 10^5^ and 5 × 10^4^ cells/mL, respectively. The cells were subjected to treatment with concentrations ranging from 12.5 to 200 µg/mL, and cell viability was assessed utilizing the MTT assay.

### 4.4. Determination of the Anti-UVB-Induced Skin Damage Effects on HaCaT Cells

To investigate the anti-UVB-stimulated skin damage effects on HaCaT cells, the cells were exposed to SHP2 at concentrations of 50, 100, and 200 µg/mL and irradiated (30 mJ·cm^−2^) with UVB. After 24 h of incubation, cell viability, intracellular ROS levels, and apoptotic body formation were assessed using the MTT assay, DCFH-DA assay, and Hoechst 33342 staining, respectively [3].

### 4.5. Determination of the Anti-UVB-Induced Skin Damage Effects on HDF Cells

HDF cells were treated with SHP2 at concentrations of 50, 100, and 200 µg/mL and then irradiated with UVB (50 mJ/cm^2^) to assess the protective effects of SHP2 against UVB-induced skin damage. Intracellular ROS levels and cell viability were evaluated using the DCFH-DA and MTT assays, respectively. To analyze collagen synthesis and MMP expression levels, SHP2-treated HDF cells were irradiated with UVB (50 mJ/cm^2^) and incubated in FBS-free media for 48 h. The cell culture media were collected and used to assess MMPs expression and PIP levels, which reflect collagen synthesis. Collagen synthesis in HDF cells was evaluated using an ELISA kit (Cat. # MK101), while MMP expression levels were measured using Sigma’s Sandwich ELISA kits, following the manufacturers’ instructions [33].

### 4.6. Western Blotting

For Western blotting, cell lysates were prepared using RIPA buffer containing protease and phosphatase inhibitors. The protein concentration was measured using a BCA assay, and the sample concentrations were adjusted to ensure equal loading. Electrophoresis was performed at 80–120 V. The proteins were transferred onto nitrocellulose membranes using a transfer system at 90 V for 90 min. The membrane was then blocked with 5% skim milk in TBST and incubated with the primary antibody overnight at 4 °C, followed by incubation with an HRP-conjugated secondary antibody for 2 h. Protein bands were detected using ECL reagent, and images were captured using a Fusion Solo system (Vilber Lourmat, Collégien, France).

### 4.7. In Vivo Studies in Zebrafish

Adult zebrafish were maintained according to the protocols outlined in our previous study [3]. The protective effect of SHP2 against UVB was investigated using 2-day post-fertilization (2dpf) larvae by treating with SHP2 (50, 100, and 200 µg/mL). After a treatment duration of 1 h, the larvae were exposed to UVB radiation at a dose of 50 mJ·cm^−2^ and incubated for 6 h. ROS generation, cell death, and lipid peroxidation were assessed using DCFH-DA, acridine orange, and DPPP, respectively [34]. 

### 4.8. Statistical Analysis

All experiments were performed in triplicate. Data are presented as the mean ± standard error (SE). Statistical analysis was conducted using SPSS 20.0, employing a one-way ANOVA to compare the mean values. Significant differences between means were determined using Tukey’s test.

## 5. Conclusions

In this study, we investigated the antioxidant and photoprotective properties of SHP2 against UVB-induced photodamage, utilizing both in vitro and in vivo models. These results indicate that SHP2 purified from *H. abdominalis* had a protective effect against UVB-induced oxidative stress in both HaCaT and HDF cells. SHP2 increased collagen synthesis by suppressing oxidative stress and inhibiting MMP expression in HDF cells, demonstrating a protective effect against UVB dermal damage. SHP2 treatment restored the expression of key antioxidant proteins such as HO-1, Nrf2, and SOD while reducing Keap1 expression in UVB-irradiated HDF cells. These results confirm the role of SHP2 in modulating the Keap1/Nrf2/HO-1 signaling pathway, contributing to its anti-photoaging activity. Furthermore, SHP2 inhibits UVB-induced damage in zebrafish by reducing cell death, lipid peroxidation, and ROS levels. Overall, this study indicates that SHP2 has anti-photoaging activity. However, a limitation of this study is the need for further exploration of SHP2’s long-term effects in other in vivo and in vitro biological systems, as well as its potential mechanisms of action, to confirm its viability as a functional ingredient in cosmetics.

## Data Availability

Data are contained within the article.
